# Classification methods for the development of genomic signatures from high-dimensional data

**DOI:** 10.1186/gb-2006-7-12-r121

**Published:** 2006-12-20

**Authors:** Hojin Moon, Hongshik Ahn, Ralph L Kodell, Chien-Ju Lin, Songjoon Baek, James J Chen

**Affiliations:** 1 Division of Biometry and Risk Assessment, National Center for Toxicological Research, FDA, NCTR Road, Jefferson, AR 72079, USA; 2 Department of Applied Mathematics and Statistics, Stony Brook University, Stony Brook, NY 11794-3600, USA

## Abstract

Personalized medicine is defined by the use of genomic signatures of patients to assign effective therapies. We present Classification by Ensembles from Random Partitions (CERP) for class prediction and apply CERP to genomic data on leukemia patients and to genomic data with several clinical variables on breast cancer patients. CERP performs consistently well compared to the other classification algorithms. The predictive accuracy can be improved by adding some relevant clinical/histopathological measurements to the genomic data.

## Abstract 2

Several classification algorithms for class prediction using high-dimensional biomedical data are presented and applied to data from leukaemia and breast cancer patients

## Background

Providing guidance on specific therapies for pathologically distinct tumor types to maximize efficacy and minimize toxicity is important for cancer treatment [1,2]. For acute leukemia, for instance, different subtypes show very different responses to therapy, reflecting the fact that they are molecularly distinct entities, although they have very similar morphological and histopathological appearance [1]. Thus, accurate classification of tumor samples is essential for efficient cancer treatment on a target population of patients. Microarray technology has been increasingly used in cancer research because of its potential for classification of tissue samples based only on gene expression data, without prior and often subjective biological knowledge [1,3,4]. Much research involving microarray data analysis is focused on distinguishing between different cancer types using gene expression profiles from disease samples, thereby allowing more accurate diagnosis and effective treatment of each patient.

Gene expression data might also be used to improve disease prognosis in order to prevent some patients from having to undergo painful unsuccessful therapies and unnecessary toxicity. For example, adjuvant chemotherapy for breast cancer after surgery could reduce the risk of distant metastases; however, seventy to eighty percent of patients receiving this treatment would be expected to survive metastasis-free without it [5,6]. The strongest predictors for metastases, such as lymph node status and histological grade, fail to classify accurately breast tumors according to their clinical behavior [6,7].

Predicting patient response to therapy or the toxic potential of drugs based on high-dimensional data are common goals of biomedical studies. Classification algorithms can be used to process high-dimensional genomic data for better prognostication of disease progression and better prediction of response to therapy to help individualize clinical assignment of treatment. The predictive models built are required to be highly accurate, since the consequence of misclassification may result in suboptimal treatment or incorrect risk profile. Commonly, there are numerous genomic and clinical predictor variables over a relatively small number of patients for biomedical applications, which presents challenges for most traditional classification algorithms to avoid over-fitting the data.

Class prediction is a supervised learning method where the algorithm learns from a training set (known samples) and establishes a prediction rule to classify new samples. Development of a class prediction algorithm generally consists of three steps: first, selection of predictors; second, fitting the prediction model to develop the classification rule; and third, performance assessment. The first two steps build a prediction model, and the third step assesses the performance of the model. Some classification algorithms, such as the classification tree or stepwise logistic regression, perform the first two steps simultaneously. Sensitivity (SN) and specificity (SP) as well as positive predictive value (PPV) and negative predictive value (NPV) are primary criteria used in the evaluation of the performance of a classification algorithm. The SN is the proportion of correct positive classifications out of the number of true positives. The SP is the proportion of correct negative classifications out of the number of true negatives. The accuracy is the total number of correct classifications out of the total number of samples. The PPV is the probability that a patient is positive given a positive prediction, while the NPV is the probability that a patient is negative given a negative prediction. Algorithms with high SN and high SP as well as high PPV and high NPV, which will have high accuracy, are obviously desirable.

Recently, a new ensemble-based classification algorithm, Classification by Ensembles from Random Partitions (CERP) has been developed [8]. This algorithm is designed specifically for high-dimensional data sets. Rationales behind CERP are as follows: first, multiple classifiers can capture most aspects of the underlying biological phenomena encoded in the data; and second, combining results of multiple diversified models can produce a superior classifier for biomedical decision making. In this paper, we use Classification-Tree CERP (C-T CERP), which is an ensemble of ensembles of optimal classification trees based on the Classification and Regression Trees (CART) algorithm [9], constructed with randomly partitioned mutually exclusive subsets from the entire predictor set. The number of features in each subset is as close to equal as possible.

The performance of CERP is compared to other well-known classification algorithms: Random Forest (RF) [10], Boosting [11,12], Support Vector Machine (SVM) [13], Diagonal Linear Discriminant Analysis (DLDA) [3], Shrunken Centroids (SC) [14], CART, Classification Rule with Unbiased Interaction Selection and Estimation (CRUISE) [15], and Quick, Unbiased and Efficient Statistical Tree (QUEST) [16]. CERP utilizes a partitioning scheme to establish mutually exclusive subsets of the predictors. On the other hand, RF takes bootstrap samples of patients for each tree and randomly selects predictors with replacement from the entire set of predictors at each node. Boosting gives extra weight to previously misclassified samples. Like CERP, RF and Boosting are ensemble classifiers. SVM is a kernel-based machine learning approach. DLDA is a classification rule based on a linear discriminant function. SC is based on an enhancement of the simple nearest centroid classifier. CART, CRUISE and QUEST are single optimal trees. Among these single-tree algorithms, CART and QUEST yield binary trees and CRUISE yields multiway splits.

In this study, the classification algorithms are applied to three popular public data sets relevant to personalized medicine. The algorithms are first used for the prediction of leukemia subtypes, acute lymphoblastic leukemia (ALL) or acute myeloid leukemia (AML), based on gene-expression data [1]. They are then used on two different data sets [6,17] to predict which breast cancer patients would benefit from adjuvant chemotherapy based on gene-expression data. We also investigate if addition of seven more clinical/histopathological variables, including age, tumor size, tumor grade, angioinvasion, estrogen receptor status, progesterone receptor status and lymphocytic infiltrate, to the high-dimensional genomic data on breast cancer patients [6] enhances classification accuracy. The performance of the classification algorithm is assessed by 20 replications of 10-fold cross-validation (CV).

## Results

### Leukemia classification

Determination of cancer type and stage is often crucial to the assignment of appropriate treatment [1]. Because chemotherapy regimens for patients with ALL are different from regimens for patients with AML, distinguishing between leukemia subtypes (ALL or AML) is critical for personalized treatment. Golub *et al*. [1] described a generic approach to cancer classification of the two subtypes of acute leukemia based on gene expression monitoring by DNA microarray technology. The data set consists of 47 patients with ALL and 25 patients with AML. The gene expression levels were measured by Affymetrix high-density oligonucleotide arrays containing 6,817 human genes. Before performing normalization, the data were preprocessed by the following steps: thresholding, with a floor of 100 and ceiling of 16,000; filtering, with exclusion of genes with max/min ≤5 or (max - min) ≤500, where max and min refer to the maximum and minimum expression levels of a particular gene across 72 mRNA samples, respectively; and base-10 logarithmic transformation. The data were then summarized by 72 mRNA samples and 3,571 genes [3].

Table 1 shows performance of classification algorithms for the leukemia data, based on 20 repetitions of 10-fold CV. All algorithms considered in this study, except single optimal trees (CART, CRUISE and QUEST), gave less than four percent error rate (mostly two to three misclassifications). Among them, CERP showed the lowest error rate of 1.4% (mostly 0 or 1 misclassification). The balance between sensitivity and specificity of CERP, RF, AdaBoost, DLDA and SC algorithms was excellent; all sensitivities and specificities were above 95%. The PPV and NPV of CERP, RF, SVM and DLDA were all higher than 95%. CERP performs slightly better than the other classification algorithms used on the leukemia data set. CERP misclassified only one out of 72 samples on the average in the 20 replications of 10-fold CV. Among single optimal trees, CRUISE and QUEST gave lower error rates (less than 14%) and higher PPV (>82%). The balance between SN and SP was good among single optimal trees considered.

### Breast cancer classification

The objective of two studies [6,17] was to use gene expression data to identify patients who might benefit from adjuvant chemotherapy according to prognostication of distant metastases for breast cancer. The van 't Veer *et al*. data [6] contains 78 primary breast cancers (34 from patients who developed distant metastases within 5 years (poor prognosis) and 44 from patients who continue to be disease-free (good prognosis) after a period of at least 5 years). These samples have been selected from patients who were lymph node negative and under 55 years of age at diagnosis. Out of approximately 25,000 gene expression levels, about 5,000 significantly regulated genes (at least a two-fold difference and a *p *value of less than 0.01) in more than 3 tumors out of 78 were selected [6]. In addition, seven relevant clinical/histopathological predictors were added to this gene expression data to investigate if the addition of these variables improves the prediction accuracy compared to genomic data only.

In the study of van de Vijver *et al*. [17], there was a cohort of young women with stage I or II breast cancer who were treated at the hospital of the Netherlands Cancer Institute. They were younger than 53 years old, 151 of whom were lymph-node-negative and 144 of whom were lymph-node-positive. Among 295 patients, 180 had a poor-prognosis signature and 115 had a good-prognosis signature. From approximately 25,000 human genes, we selected about 5,000 genes according to correlation of the microarray data with the prognosis profile [17]. There were no missing data.

Tables 2 and 3 show performance of classification algorithms for the van 't Veer *et al*. [6] breast cancer genomic data and genomic plus clinical/histopathological data, respectively, based on 20 repetitions of 10-fold CV. When seven more clinical variables are added to the gene expression data, the prediction accuracy appears to be slightly improved compared to accuracies from genomic data only. This is mainly due to an improvement in sensitivity. Still, the overall accuracy is somewhat low for all the classifiers. The balance between SN and SP is reasonably good for CERP, DLDA and SC. Sensitivities of CERP, DLDA and SC are higher (>50%) than the rest (<50%). The positive predictive values from CERP, RF, AdaBoost, DLDA and SC are higher (>55%) than the others. Among single optimal trees, accuracies of CRUISE and QUEST are slightly higher than CART (>55%). However, the balance between SN and SP in these single trees is unsatisfactory.

**Figure 1:**
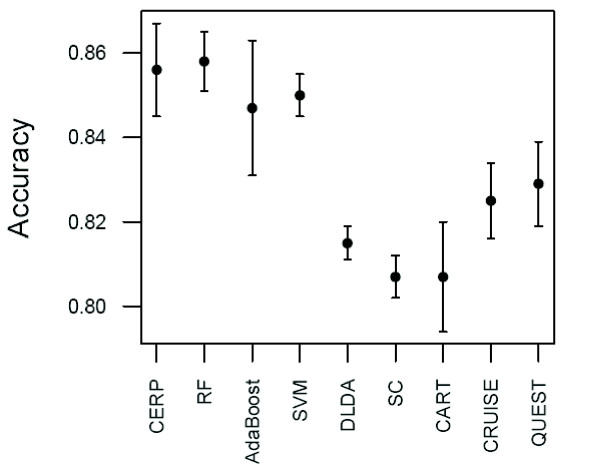
Accuracy of classification algorithms for the van de Vijver *et al*. [17] data.

Figure 1 shows the accuracies of classification algorithms for the van de Vijver *et al*. data [17] based on 20 repetitions of 10-fold CV. The overall accuracy is improved and greater than 80% for all the classification algorithms compared to accuracies from the van 't Veer *et al*. [6] data. Among the algorithms, the accuracies of CERP, RF and SVM are greater than 85%. The balance between SN and SP (not shown) is slightly better for CERP (SN 87.5%, and SP 82.5%) than RF (SN 89.1% and SP 80.7%) and SVM (SN 89.1% and SP 78.7%). The balance between positive and negative predictive values (not shown) from CERP, RF and SVM are better than those from the others (PPV and NPV >80%).

## Discussion

Recent advancements in biotechnology have accelerated research on the development of molecular biomarkers for the diagnosis and treatment of disease. The Food and Drug Administration envisions clinical pharmacogenomic profiling to identify patients most likely to benefit from particular drugs and patients most likely to experience adverse reactions. Such patient profiling will enable assignment of drug therapies on a scientifically sound predictive basis rather than on an empirical trial-and-error basis. The goal is to change medical practice from a population-based approach to an individualized approach.

We have presented statistical classification algorithms to accurately classify patients into risk/benefit categories using high-dimensional genomic and other data. Classification algorithms were illustrated by three published data sets and the new C-T CERP was compared to the best known published classification procedures. CERP is a consistently good algorithm and maintains a good balance between sensitivity and specificity even when sample sizes between classes are unbalanced.

In one application, leukemia patients were classified as having either ALL or AML based on each individual patient's gene-expression profile. The distinction is important because the chemotherapies required for the two subtypes are very different, and incorrect treatment assignment has both efficacy and toxicity consequences. Classification algorithms are essential for the realization of personalized medicine in this application, because distinguishing ALL and AML otherwise requires an experienced hematologist's interpretation of several analyses performed in a highly specialized laboratory. CERP correctly classified patients with the lowest cross-validated error rate of 1.4% (0 or 1 misclassification) compared to the other classification procedures we considered (more than 1 misclassification). This level of accuracy shows the real potential for confident clinical assignment of therapies on an individual patient basis.

In the other application, post-surgery breast cancer patients were classified by the algorithms as having either a good or poor prognosis, in terms of the likelihood of distant metastasis within five years, based on gene-expression profiles. If this were brought into clinical application, a patient with a confidently predicted good prognosis might want to elect out of adjuvant chemotherapy and its associated debilitating side effects. With current rule-based decisions, almost all patients are subjected to chemotherapy. When just a few clinical and histopathological measures traditionally used for treatment assignment were added to the numerous genomic predictors, the prediction accuracy appeared to be enhanced further. According to the theory underlying the CERP algorithm, importantly, the more individual patient information that is used, whatever the source or type, the greater is the likelihood that the prediction accuracy will increase. While the van 't Veer *et al*. data [6] do not contain enough information to allow confident prognoses, the van de Vijver *et al*. data [17] show improved cross-validated overall accuracy that might be sufficiently high for clinical practice. It is worth noting that CERP and all the other methods do not perform as well as the method reported in the van 't Veer *et al*. [6] study (62.3% versus 83% accuracy). It may be that the feature selection method used by van 't Veer *et al*. overfit the data and they did have a true cross-validation test. They appear to have used correlation with outcome for feature selection outside the cross-validation procedure. It is anticipated that the combined use of multiple biomarkers on individual patients could improve the prediction accuracy of data like the present genomic data to a level suitable for clinical practice.

## Materials and methods

### Ensemble methods to enhance prediction accuracy

Let *X*_*i *_be a random variable indicating a classification by the *i*-th independent classifier, where *X*_*i *_= 1 if the classification is correct and *X*_*i *_= 0 if not. We let *p *be the prediction accuracy of each classifier. Then the *X*_*i *_are Bernoulli(*p*), and the number of accurate classifications by the ensemble majority voting method is:

Y=∑i=1rXi,
 MathType@MTEF@5@5@+=feaafiart1ev1aaatCvAUfeBSjuyZL2yd9gzLbvyNv2Caerbhv2BYDwAHbqedmvETj2BSbqee0evGueE0jxyaibaiKI8=vI8tuQ8FMI8Gi=hEeeu0xXdbba9frFj0=OqFfea0dXdd9vqai=hGuQ8kuc9pgc9s8qqaq=dirpe0xb9q8qiLsFr0=vr0=vr0dc8meaabaqaciGacaGaaeqabaqadeqadaaakeaacaWGzbGaeyypa0ZaaabmaeaacaWGybWaaSbaaSqaaiaadMgaaeqaaaqaaiaadMgacqGH9aqpcaaIXaaabaGaamOCaaqdcqGHris5aOGaaiilaaaa@3D5F@

which is Binomial(*r*, *p*). We let *r *= 2*k *+ 1, where *k *is a nonnegative integer. We define the prediction accuracy of the ensemble by majority voting as:

*A*_*r *_= *P*(*Y *≥ *k *+ 1).

Then the prediction accuracy of the ensemble can be obtained using the standard binomial probability:

Ar=∑i=k+1r(ri)pi(1−p)r−i.
 MathType@MTEF@5@5@+=feaafiart1ev1aaatCvAUfeBSjuyZL2yd9gzLbvyNv2Caerbhv2BYDwAHbqedmvETj2BSbqee0evGueE0jxyaibaiKI8=vI8tuQ8FMI8Gi=hEeeu0xXdbba9frFj0=OqFfea0dXdd9vqai=hGuQ8kuc9pgc9s8qqaq=dirpe0xb9q8qiLsFr0=vr0=vr0dc8meaabaqaciGacaGaaeqabaqadeqadaaakeaacaWGbbWaaSbaaSqaaiaadkhaaeqaaOGaeyypa0ZaaabmaeaadaqadaqaauaabeqaceaaaeaacaWGYbaabaGaamyAaaaaaiaawIcacaGLPaaaaSqaaiaadMgacqGH9aqpcaWGRbGaey4kaSIaaGymaaqaaiaadkhaa0GaeyyeIuoakiaadchadaahaaWcbeqaaiaadMgaaaGccaGGOaGaaGymaiabgkHiTiaadchacaGGPaWaaWbaaSqabeaacaWGYbGaeyOeI0IaamyAaaaakiaac6caaaa@4AF0@

It has been shown that the majority vote is guaranteed to give a higher accuracy than an individual classifier when the individual classifiers have an accuracy greater than 0.5 [8]. In practice, the classifiers may be correlated to a certain degree. When classifiers are positively correlated, they tend to produce the same prediction outcomes. Kuncheva *et al*. [18] relaxed the restriction that the classifiers be independent. When the classifiers in the ensemble are positively correlated, we use the beta-binomial model [19-21] to obtain the prediction accuracy. The beta-binomial model is commonly used to model positively correlated binary variables.

Table 4 illustrates the theoretical prediction accuracy obtained by ensemble majority voting. The table illustrates that independent classifiers improve the prediction accuracy more rapidly than the correlated classifiers. For example, when the prediction accuracy of each base classifier is 80%, the class prediction accuracy by the majority vote in an ensemble reaches nearly 100% with *r *= 25 independent classifiers. On the other hand, the accuracy of the majority vote reaches only 87.7% with *r *= 101 positively correlated classifiers (the correlation ρ = 0.3). These results imply that the prediction accuracy of the ensemble majority vote will increase by adding more classifiers. However, if the classifiers are highly positively correlated, the addition will not help much to increase the prediction accuracy. CERP uses random partitioning to create mutually exclusive subsets of the features to introduce diversity. If the number of partitions is larger, the prediction accuracy of the individual classifier would be lower. To compensate for this loss, new ensembles are added. When the classifiers are negatively correlated, the prediction accuracy improves more rapidly than with independent classifiers. Ahn *et al*. [8] reported a theoretical result showing enhancement of the prediction accuracy by ensemble majority voting of negatively correlated classifiers.

**Figure 2:**
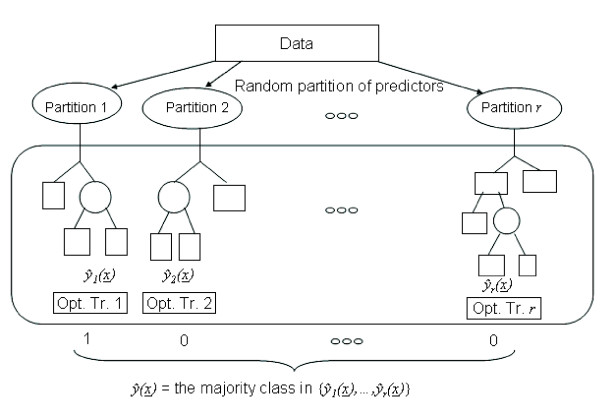
An ensemble in CERP.

Figure 2 shows a schematic diagram of an ensemble of CERP. Predictor variables in a data set are randomly subdivided into *r *mutually exclusive subsets. In this study, we partitioned the feature space such that each subspace contains approximately *n*/6 predictors. Predictor variables in a data set are randomly subdivided into *r *mutually exclusive subsets by shuffling the features, where *r *= 6*m*/*n*. For example, in the leukemia data set, there are *m *= 3,571 features, *n *= 72 samples, and *r *= 6 × 3,571/72 = 297 subsets. Each subset has 72/6 = 12 or 13 features. Using the *i*-th subset of predictors, a tree is constructed under the Gini diversity index measure [9]. This tree construction process for growing a large initial tree continues splitting the samples until either each terminal node is pure (that is, the node cases are all in one class) or the total number of samples in a node is ≤5. To avoid over-fitting, the optimal trees in C-T CERP are obtained by employing the minimal cost-complexity pruning algorithm used in CART. In the pruning process, a nested sequence of subtrees is obtained by progressively deleting branches. This results in a decreasing sequence of subtrees in terms of tree complexity. One of these subtrees is selected as an optimal tree if a subtree produces a minimal internal cross-validated misclassification error within 1-SE [9].

In C-T CERP, we employ majority voting among trees within individual ensembles and then among ensembles. In an ensemble, using training data, only the trees that have highest sensitivity and specificity (>90%) are kept, which reduces each ensemble down to a small number of tree classifiers. When the selected trees are less than three in an ensemble, the cut-off value is decreased by five percent increments until at least three trees are selected. New ensembles are created by randomly re-partitioning the feature space and similarly reducing to a different set of classifiers. Most of the improvement in adding ensembles was achieved by the first few ensembles, and then the improvement was slowed down as more ensembles were added [8]. In this paper, we fixed the default number of ensembles as 15 according to our preliminary results. Final ensemble prediction is then based on the majority vote across these ensembles. C-T CERP is implemented in C. A potential user can obtain the software by contacting the authors or by downloading from the worldwide web site [22].

A package (RandomForest) in R is used for the RF algorithm. The number of trees is generated using the default of *ntree = 500*. The number of features selected at each node in a tree is selected using the default value of *floor*(*m*^1/2^), where *m *is the total number of features. Similarly, a package (e1071) in R is applied for the SVM, in which radial basis kernel is used as a default. Among many boosting methods, AdaBoost [11] is adopted using a package (boost) in R with a default option. For DLDA, a package (sma) in R is employed with a default option. SC is implemented with a package (pamr) in R with a soft thresholding option as a default. For single optimal trees, CART is implemented with a package (rpart) in R with a default option. On the other hand, compiled binaries are downloaded from the website [23], and implemented in R for CRUISE and QUEST.

In many cases, the number of features (*m*) is much greater than the number of patients (*n*). In such a case, cross-validation is used to obtain a valid measure of prediction accuracy for genomic signature classifiers. CV utilizes resampling without replacement of the entire data set to repeatedly develop classifiers on a training set and evaluates classifiers on a separate test set, and then averages the procedure over the resamplings.

We evaluated the prediction accuracy, the balance between sensitivity (SN) and specificity (SP), and the balance between positive predictive value (PPV) and negative predictive value (NPV) of the classification algorithms considered by averaging the results from 20 replications of 10-fold CV in order to achieve a stable result. Twenty CVs should be sufficient according to Molinaro *et al*. [24] who recommended ten trials of ten-fold CV to have low MSE and bias.

## Figures and Tables

**Table 1:** Performance of classification algorithms for the leukemia data based on 20 repetitions of 10-fold CV

Algorithm	Accuracy	Sensitivity*	Specificity^†^	PPV^‡^	NPV^§^
CERP	98.6 (<.001)	96.0 (<.001)	100.0 (.000)	100.0 (.000)	97.9 (<.001)
RF	97.9 (.008)	95.0 (.022)	99.5 (.009)	99.0 (.018)	97.4 (.011)
AdaBoost	96.0 (.005)	95.6 (.012)	96.3 (.009)	93.2 (.016)	97.6 (.006)
SVM	97.2 (.012)	92.0 (.034)	100.0 (.000)	100.0 (.000)	95.9 (.017)
DLDA	97.5 (.007)	96.0 (<.001)	98.3 (.011)	96.8 (.021)	97.9 (<.001)
SC	96.0 (.004)	96.0 (<.001)	96.0 (.007)	92.7 (.011)	97.8 (<.001)
CART	81.7 (.035)	76.2 (.046)	84.6 (.053)	72.4 (.067)	87.0 (.021)
CRUISE	86.8 (.021)	79.8 (.040)	90.5 (.029)	82.0 (.044)	89.4 (.018)
QUEST	86.9 (.020)	79.4 (.042)	91.0 (.032)	82.7 (.048)	89.3 (.018)

SD is given in parentheses. *AML considered positive. ^†^ALL considered negative. ^‡^Positive predictive value. ^§^Negative predictive value.

**Table 2:** Performance of classification algorithms for the van 't Veer *et al*. breast cancer genomic data based on 20 repetitions of 10-fold CV

Algorithm	Accuracy	Sensitivity*	Specificity^†^	PPV^‡^	NPV^§^
CERP	62.3 (.023)	50.9 (.037)	71.1 (.026)	57.7 (.029)	65.2 (.020)
RF	62.5 (.019)	46.8 (.032)	74.7 (.032)	58.9 (.029)	64.5 (.014)
AdaBoost	58.8 (.041)	32.1 (.089)	79.4 (.069)	55.0 (.094)	60.3 (.028)
SVM	56.5 (.029)	39.6 (.053)	69.7 (.027)	50.1 (.042)	59.9 (.025)
DLDA	62.5 (.019)	52.4 (.023)	70.3 (.026)	57.8 (.026)	65.6 (.015)
SC	60.9 (.019)	50.6 (.026)	68.9 (.023)	55.7 (.024)	64.3 (.016)
CART	54.6 (.028)	17.5 (.058)	83.2 (.047)	44.6 (.084)	56.6 (.018)
CRUISE	55.1 (.048)	21.5 (.100)	81.0 (.059)	45.6 (.112)	57.3 (.034)
QUEST	56.5 (.044)	22.8 (.080)	82.6 (.077)	51.0 (.117)	58.1 (.027)

SD is given in parentheses. *Poor prognosis considered positive. ^†^Good prognosis considered negative. ^‡^Positive predictive value. ^§^Negative predictive value.

**Table 3:** Performance of classification algorithms for the van 't Veer *et al*. breast cancer genomic and clinical/histopathological data based on 20 trials of 10-fold CV

Algorithm	Accuracy	Sensitivity^*^	Specificity^†^	PPV^‡^	NPV^§^
CERP	63.3 (.024)	52.5 (.042)	71.6 (.027)	58.8 (.031)	66.1 (.022)
RF	63.0 (.023)	48.2 (.034)	74.4 (.034)	59.4 (.034)	65.1 (.016)
AdaBoost	61.9 (.045)	38.7 (.090)	79.8 (.065)	59.9 (.085)	62.8 (.034)
SVM	57.4 (.027)	40.3 (.044)	70.7 (.037)	51.5 (.040)	60.5 (.021)
DLDA	62.9 (.017)	52.6 (.025)	70.9 (.027)	58.4 (.023)	66.0 (.013)
SC	62.2 (.018)	53.8 (.025)	68.8 (.018)	57.1 (.022)	65.8 (.016)
CART	54.7 (.031)	21.6 (.096)	80.3 (.063)	44.3 (.103)	57.2 (.022)
CRUISE	57.5 (.047)	24.0 (.100)	83.4 (.063)	51.9 (.120)	58.8 (.032)
QUEST	56.3 (.036)	21.8 (.062)	83.1 (.071)	50.7 (.082)	57.8 (.021)

SD is given in parentheses. *Poor prognosis considered positive. ^†^Good prognosis considered negative. ^‡^Positive predictive value. ^§^Negative predictive value.

**Table 4:** Enhancement of the prediction accuracy by ensemble majority voting*

*r*	ρ	Prediction accuracy of each base classifier				
		0.5	0.6	0.7	0.8	0.9
3	0	0.5	0.648	0.784	0.896	0.972
	0.1	0.5	0.635	0.762	0.871	0.953
	0.3	0.5	0.618	0.732	0.836	0.927
15	0	0.5	0.787	0.950	0.996	1.000
	0.1	0.5	0.695	0.851	0.947	0.990
	0.3	0.5	0.636	0.762	0.868	0.948
25	0	0.5	0.846	0.986	1.000	1.000
	0.1	0.5	0.708	0.868	0.958	0.993
	0.3	0.5	0.639	0.766	0.872	0.951
101	0	0.5	0.980	1.000	1.000	1.000
	0.1	0.5	0.728	0.891	0.971	0.996
	0.3	0.5	0.642	0.771	0.877	0.954

*Binomial probability used for ρ = 0, with normal approximation for *r *> 25; Beta-binomial probability used for ρ > 0.
